# Identifying and Describing the Impact of Cyclone, Storm and Flood Related Disasters on Treatment Management, Care and Exacerbations of Non-communicable Diseases and the Implications for Public Health

**DOI:** 10.1371/currents.dis.62e9286d152de04799644dcca47d9288

**Published:** 2015-09-28

**Authors:** Benjamin Ryan, Richard C. Franklin, Frederick M. Burkle, Peter Aitken, Erin Smith, Kerrianne Watt, Peter Leggat

**Affiliations:** College of Public Health, Medical and Veterinary Sciences, James Cook University, Australia; Cairns and Hinterland Hospital and Health Service, Australia; College of Public Health, Medical and Veterinary Sciences, James Cook University, Cairns, Queensland, Australia; Harvard Humanitarian Initiative, Harvard University, Cambridge, Massachusetts; The Woodrow Wilson International Center for Scholars, Washington, DC, USA; College of Public Health, Medical and Veterinary Sciences, James Cook University, Australia; School of Public Health, Queensland University of Technology, Australia; School of Medical Sciences, Edith Cowan University; College of Public Health, Medical and Veterinary Sciences, James Cook University, Townsville, Queensland, Australia; College of Public Health, Medical and Veterinary Sciences, James Cook University, Australia; World Safety Organization Collaborating Centre for Injury Prevention and Safety Promotion; College of Public Health, Medical and Veterinary Sciences, James Cook University, Townsville, Queensland, Australia; Faculty of Health Sciences, Flinders University, Adelaide, South Australia, Australia

## Abstract

**Introduction:** Over the last quarter of a century the frequency of natural disasters and the burden of non-communicable diseases (NCD) across the globe have been increasing. For individuals susceptible to, or chronically experiencing, NCDs this has become a significant risk. Disasters jeopardize access to essential treatment, care, equipment, water and food, which can result in an exacerbation of existing conditions or even preventable death. Consequently, there is a need to expand the public health focus of disaster management to include NCDs. To provide a platform for this to occur, this article presents the results from a systematic review that identifies and describes the impact of cyclone, flood and storm related disasters on those susceptible to, or experiencing, NCDs. The NCDs researched were: cardiovascular diseases; cancers; chronic respiratory diseases; and diabetes.

**Methods:** Four electronic publication databases were searched with a date limit of 31 December 2014. The data was analyzed through an aggregation of individual papers to create an overall data description. The data was then grouped by disease to describe the impact of a disaster on treatment management, exacerbation, and health care of people with NCDs. The PRISMA checklist was used to guide presentation of the research.

**Results:**  The review identified 48 relevant articles. All studies represented developed country data. Disasters interrupt treatment management and overall care for people with NCDs, which results in an increased risk of exacerbation of their illness or even death. The interruption may be caused by a range of factors, such as damaged transport routes, reduced health services, loss of power and evacuations. The health impact varied according to the NCD. For people with chronic respiratory diseases, a disaster increases the risk of acute exacerbation. Meanwhile, for people with cancer, cardiovascular diseases and diabetes there is an increased risk of their illness exacerbating, which can result in death.

**Conclusion:**  Cyclone, flood and storm related disasters impact on treatment management and care for people with NCDs. Possible consequences include exacerbation of illness, complications or even death. There is now a need to expand traditional disaster approaches by public health to incorporate NCDs. This must be guided by the major NCDs identified by the World Health Organization and implemented in-line with the Sendai Framework for Disaster Risk Reduction: 2015-2030. This includes understanding all the factors that influence both direct and indirect (preventable) morbidity and mortality related to NCDs during and after disasters. Once achieved, disaster planners and public health professionals will be in a position to develop and implement effective mitigation strategies.

## Introduction

Over the past quarter of century there has been a global increase in the frequency and severity of disasters and burden of non-communicable diseases (NCD).^1^^,^ 2 For example, since 2005 disasters have resulted in over 700 thousand deaths, over 1.4 million people being injured, approximately 23 million people homeless, 1.5 billion people affected and economic losses of more than $1.3 trillion.^3^ Of the disasters worldwide cyclone, flood or storms have accounted for 88% and are responsible for 76% of disaster related deaths.^4^^,^ 5 This threat is anticipated to continue, if not increase, with climate change expected to make extreme weather events, such as cyclones, floods and storms, more frequent and severe.^3^^,^ 6 ^,^ 7 For individuals with NCDs this represents a significant risk because disasters jeopardize access to and often availability of essential public health treatment options, equipment, clean water and food, which can result in an exacerbation of existing conditions or even preventable death.^1^^,^ 8 ^,^ 9 ^,^ 10 ^,^ 11 ^,^ 12 ^,^ 13 ^,^ 14

This problem has been recognized globally by the United Nations in the Sendai Framework for Disaster Risk Reduction: 2015-2030 (Sendai Framework). Item 30(k) relates to chronic diseases (interchangeable with NCDs) and requests that due to their particular needs should be included in the design of policies and plans to manage risks before, during and after disasters, including having access to life-saving services.^3^ This call to action builds on and compliments the World Health Organization (WHO) Global Action Plan for the Prevention and Control of Noncommunicable Diseases – 2013-2020 (WHO Action Plan).^15^

NCDs are prolonged illnesses, rarely cured completely and are not passed from person to person.^16^^,^ 17 There are four major groupings: cardiovascular diseases; cancers; chronic respiratory diseases; and diabetes.^15^^,^ 17 ^,^ 18  These conditions account for 79% of NCD deaths globally and have common behavioral risk factors (smoking, physical inactivity, poor nutrition and harmful use of alcohol).^15^^,^ 17 ^,^ 18 Minor groupings within NCDs include arthritis, obesity, mental health and renal conditions.^16^^,^ 17 ^,^ 18 ^,^ 19

The risk disasters pose to people with NCDs is further highlighted by the traditional public health focus on communicable diseases following a disaster when the actual risk is low (particularly in developed countries).^20^ Improvements in life expectancy along with changes in lifestyle and diet have contributed to a ‘disease transition’ from communicable diseases to NCDs.^1^^,^ 17 ^,^ 21 ^,^ 22 NCDs are costly and time exhaustive to treat, which has implications for health systems’ capacity and capability.^23^^,^ 24 This impact is expected to rise over the coming decades as the prevalence of NCDs across the world increases, public health infrastructure is further compromised and economic pressures are placed on health systems.^16^^,^ 17 ^,^ 22 ^,^ 25

To properly address and define the risk disasters pose to people with NCDs, public health focus of disaster management should be expanded to include the management of people with NCDs. To better identify the much needed administrative and operational platform for this to occur, a systematic review of the literature identifying and describing the impact of cyclone, flood and storm related disasters on people with the four major NCDs was completed. The NCDs included: cardiovascular diseases (heart attacks and stroke), cancers, chronic respiratory diseases (chronic obstructed pulmonary disease and asthma) and diabetes.^15^^,^ 17 ^,^ 18

A focus on cyclone, flood and storms is required to ensure the research reflects the natural disasters that are the most frequent and deadly across the world.^4^^,^ 5 The objectives of this research were to: determine and describe the impact on the treatment management, exacerbation of and direct care for those with NCDs; and identify the impact on the health of the at-risk population with chronic NCDs. The findings presented will help public health disaster planners and professionals understand the impact of disasters on this most vulnerable population and the impact on both direct and indirect health consequences.

## Methodology

An integrative review methodology was selected to systematically review literature due to its effectiveness in defining new concepts and direct applicability to practice and policy.^26^^,^ 27 This methodology also allows specific aspects of previous research to be critically and methodically evaluated.^28^ The PRISMA checklist was used to guide presentation of the research (Appendix 1).^29^

Consistent with an integrative review methodology the research was conducted over the stages of problem identification; literature search; data evaluation; and data analysis.^26^ The problem identification phase was incorporated into the introduction section of this paper. An overview of the remaining three stages is provided in the following.

**1. Literature search**

The literature search included three components. The first was determining the search terms, second searching databases and third applying the inclusion/exclusion criteria. This process is described in the following.

*1.1 Search terms*

The search terms were determined through a process of testing, refining and finalising in Medline between November and December 2014. The Medline database was selected because it is an essential tool for biomedical and allied health researchers and practitioners conducting literature searches.^30^^,^ 31 The search terms selected were: ‘cyclone’, ‘storm’ or ‘flood’; and ‘disaster’; and variations for ‘non-communicable disease’, ‘cancer’, ‘cardiovascular disease’, ‘chronic respiratory disease’ or ‘diabetes’. The variations included were based on World Health Organization (WHO) NCD terms and include:

- Non-communicable disease: ‘NCD’ or ‘chronic disease’.
- Cancer: ‘malignant tumours’ or ‘neoplasms’.^32^
- Cardiovascular disease: ‘coronary heart disease’, ‘cerebrovascular disease’, ‘peripheral arterial disease’, ‘rheumatic heart disease’, ‘congenital heart disease’, ‘deep vein thrombosis’ or ‘pulmonary embolism’.^33^
- Chronic respiratory diseases: ‘asthma’, ‘chronic obstructive pulmonary disease’, ‘COPD’, ‘occupational lung diseases’, ‘lung disease’ or ‘pulmonary hypertension’.^34^
- Diabetes: no variations, this term is specific for a condition where the pancreas does not produce enough insulin or when the body cannot effectively use the insulin it produce.^35^

To further maximize the search variations of NCD terms those identified by WHO were used and included: ‘COPD’ and ‘respiratory disease’ for chronic obstructive pulmonary disease; ‘lung disease’ for occupational lung diseases; and ‘heart disease’ for coronary, rheumatic and congenital heart disease. Other terms such as obesity, chronic pain and alcohol related disorders were considered, however, they were not identified as NCDs by WHO. Based on this, the final search terms used were:

*(Cyclone OR storm OR flood) AND ((non-communicable disease OR NCD OR chronic disease) OR (cancer OR malignant tumours OR neoplasms) OR (cardiovascular disease OR coronary heart disease OR cerebrovascular disease OR peripheral arterial disease OR rheumatic heart disease OR congenital heart disease OR deep vein thrombosis OR pulmonary embolism) OR (chronic respiratory diseases OR asthma OR chronic obstructive pulmonary disease OR COPD OR occupational lung diseases OR lung disease OR pulmonary hypertension) OR (Diabetes) AND (disaster).*

*1.2 Databases*

A search of CINAHL, Medline, PsycINFO, Science Direct and Scopus databases was conducted in January 2015 with a date limit of 31 December 2014. These databases were selected to maximise the literature searched and minimise the risk of missing relevant articles. To further maximise the scope of the literature examined, reference lists of obtained literature were reviewed. Google Scholar was considered as one of the databases, however, the publications are not listed in relation to quality, they are displayed in relation to visits.^36^ Also, the retrieval and record management mechanisms lack quality when compared to other databases.^37^^,^ 38 For these reasons, Google Scholar was only used to explore reference lists and citations of obtained literature.

The search strategies differed based on the database functionality (Table 1). The Medline search was limited to articles with abstracts and references, pharmacological actions, humans and core clinical journals; Science Direct to health, patient, public health, disaster, emergency and medical; and Scopus to nursing, health professions, pharmacology, toxicology and pharmaceutics. No limitations were applied to CINAHL and PsycINFO. This approach combined with the searches of reference lists and citations in Google Scholar was designed to increase accuracy and ensure the search was tailored to each database.

**Figure:**
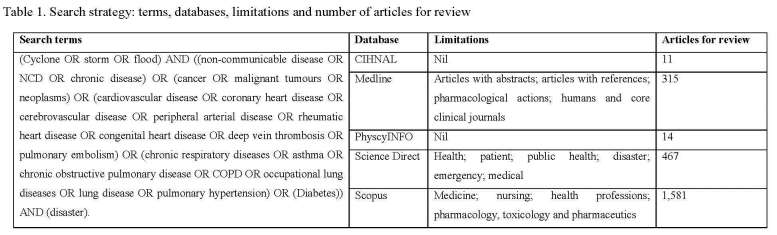

*1.3 Inclusion/exclusion criteria*

An article was considered valid if the inclusion criteria was achieved, this included: discussing how cyclone, flood or storm related disasters impacted people with NCDs. Papers were selected from peer-reviewed journal articles which were either descriptive (described a situation or a specific disaster), mixed methods, qualitative or quantitative in their methodology. Papers were excluded if they did not focus on NCDs and discussed cyclone, flood or storm related disasters. Conference abstracts and literature reviews were also excluded. The primary data sources from literature reviews were reviewed.

**2. Data evaluation**

The evaluation was conducted following the principles of qualitative research and included organizing the data, reading and memoing and data description.^39^^,^ 40 ^,^ 41 The method for each is:

- Data organisation: After being sourced the data (papers) were saved electronically and, where appropriate, hard copies stored on file.^39^
- Reading and memoing: The process was conducted through a combination of electronic notes and by hand using a highlighter and pen.^39^^,^ 40 ^,^ 41 The information gathered was captured electronically (a table embedded in a Microsoft Word ™ Document) and coded according to key phrases, ideas and concepts.^39^^,^ 40 ^,^ 41
- Data description: an individual description was developed for each paper. This included categorizing the papers into four data types: descriptive; mixed methods; qualitative; quantitative or mixed methods. This was followed by describing each paper’s key phrases, ideas, concepts and grouping papers by each NCD.^39^

**3. Data analysis**

The data was analysed through an aggregation of individual papers to create an overall data description. The descriptions of NCDs were placed in a matrix to provide an overview of the issues identified. This included columns on the impact on treatment management and care; and the health impacts of cyclone, flood and storm related disasters. This process allowed the impacts and risks to the health and well-being of people with NCDs to be systematically identified and described.

## Results

The search strategy identified 48 relevant articles. Initially 2,388 potentially relevant articles were identified. After a title and abstract review, 2,299 articles were rejected based on exclusion criteria and 89 selected for full text review. After the full text review 56 articles were rejected (including three duplicates), 33 selected for analysis and an additional six identified from the reference lists. The Google Scholar citation list for the 39 relevant articles identified an additional nine articles (Figure 1).

**Figure:**
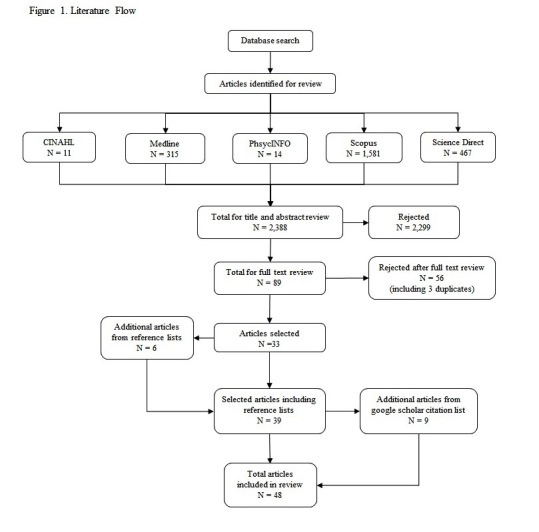

The most common data type was a quantitative article (n=24; 50%) followed by descriptive (n=21; 44%), mixed methods (n=2; 4%) and qualitative (n=1; 2%) (Appendix 2). Based on this, it can be estimated that 54% (n=27) of articles were not descriptive.

Of the 48 articles, 23 (49%) focused specifically on hurricanes, three on tsunamis (7%), two on floods (4%), two on blizzards, snowstorms or ice storms (4%) and one each (1%) for cyclone and mudslide from a rain event. The remaining 16 (33%) focused on more than one disaster type. Of the articles that focused on more than one disaster type: ten included floods, ten hurricanes, three tsunamis, three storms, two cyclones, one typhoon, one windstorm and one tornado. Other disaster types identified, which were not part of this research, were earthquake (n=7), volcano (n=2) and fire (n=1).

The most common disaster discussed was Hurricane Katrina (n=17) followed by the 2011 Japanese Tsunami (n=3) and Hurricane Iniki (n=2). Other disasters discussed (n=1) were the New York Snowstorm (1987), Maine Ice Storm (1998), mudslide due to heavy flooding in Japan in Kagoshimi Prefecture (2012), flooding in Thailand (2010) and Hurricane’s Andrew (1992), Marilyn (1995) and Sandy (2012). The remaining articles focused on the general impact of cyclone, flood and storm related disasters. The United States of America (USA) was the origin for the majority of articles (n=36, 74%), followed by Japan (n=5, 10%) and the United Kingdom (n=2, 4%). Other locations (n=1) were Australia, Denmark, Puerto Rico, South Korea and Thailand.

There were 24 different descriptions of NCDs in this research. An additional 21 conditions were also described by the 48 articles reviewed. This provided a total of 45 descriptions for NCDs (Table 2). For the NCDs targeted by this research, cardiovascular had the largest number of total descriptions (n=11) followed by chronic respiratory disease (n=8), diabetes (n=4) and cancer (n=1). All descriptions of cancer included the word cancer. The additional 15 descriptions were grouped into mental health (n=10), renal diseases (n=6) and other (n=5). The other conditions were arthritis, cystic fibrosis, HIV/AIDS, sickle cell disease and visual impairment. As mental health, renal diseases and other descriptions identified were not a focus of this research they were excluded from the analysis. This result is further explored in the discussion.

**Figure:**
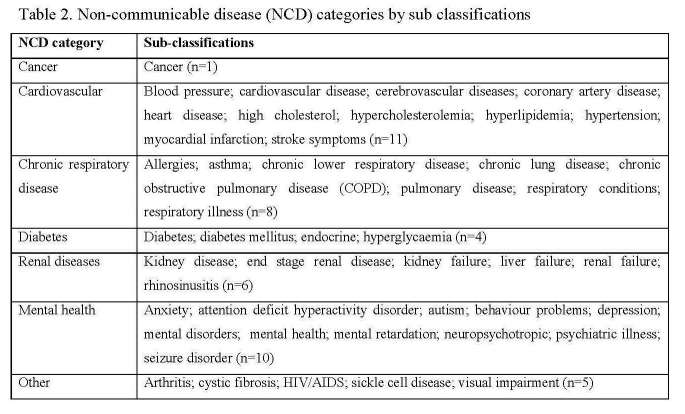

**Data Analysis - Impact, treatment management and care**

The data was analyzed according to the impact of a disaster on specific and non-specific NCDs; treatment, care and services; and the health impact (for example, exacerbation of existing NCD). The category of non-specific NCDs was used because there were a number of articles that described the impact of disasters on more than one NCD. The analysis is provided in Table 3 and described in the following.

**Figure:**
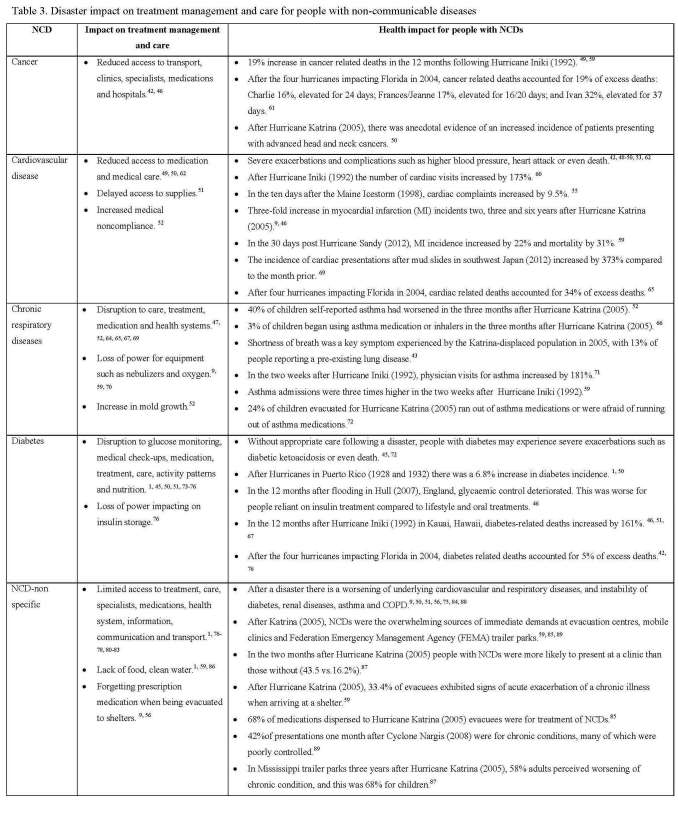

*Cancer*

For people with cancer, a disaster can reduce access to transport, clinics, specialists, medications and hospitals.^42^^,^ 43 This often results in a reduction in access to cancer treatment management and care, which based on experiences from Hurricane Katrina can last for up to one year.^44^ However, no evidence was found that a person with cancer was at risk of their illness exacerbating after a disaster. Ultimately the consequence for people with cancer is an increased risk of premature death.^45^^,^ 46

*Cardiovascular Disease*

Without appropriate care following a disaster, people with cardiovascular disease are at risk of severe exacerbation or complications of their illness such as high blood-pressure, heart attack or even death.^47^^,^ 48 ^,^ 49 ^,^ 50 ^,^ 51 ^,^ 52 ^,^ 53 ^,^ 54 ^,^ 55 ^,^ 56 ^,^ 57 ^,^ 58 ^,^ 59 This risk can continue for weeks or in the case of Hurricane Katrina years.^48^^,^ 52 ^,^ 53 ^,^ 54 ^,^ 55 ^,^ 60 This is generally due to limited access to medication, care and supplies; medical noncompliance; and the physical workload associated with clean-up and reconstruction.^1^^,^ 49 ^,^ 50 ^,^ 57 ^,^ 58 ^,^ 59 ^,^ 61 ^,^ 62 These factors culminate in people with cardiovascular diseases being at an increased risk after a disaster of severe exacerbation of their illness which can result in premature/preventable death.^46^^,^ 60

*Chronic Respiratory Disease*

There is an increased risk of people with chronic respiratory diseases having acute exacerbations after a disaster.^46^^,^ 47 ^,^ 60 ^,^ 63 ^,^ 64 ^,^ 65 There is often an increase in physician visits and hospital admissions related to chronic respiratory diseases after a disaster.^52^^,^ 66 These exacerbations are due to a disruption in care, treatment, medication, supplies, equipment, loss of power (particularly for oxygen and nebulizer dependent patients) and overcrowding in shelters.^47^^,^ 59 ^,^ 64 ^,^ 65 ^,^ 67 ^,^ 68 ^,^ 69 The high levels of mold and other allergens after a disaster are other factors increasing the risk of acute exacerbation.^70^ The review confirmed that people with chronic respiratory diseases are at an increased risk of death after a disaster.

*Diabetes*

Without appropriate care following a disaster, people with diabetes are at risk of severe exacerbations or even death all of which are preventable.^1^^,^ 45 ^,^ 50 ^,^ 59 ^,^ 71 ^,^ 72 ^,^ 73 This risk can continue for months following the event.^71^ This is due to disrupted treatment, poor nutrition, loss of power for insulin storage, limited physical activity, damaged medication, lost prescriptions and disrupted activity patterns.^43^^,^ 45 ^,^ 68 ^,^ 71 ^,^ 72 ^,^ 74 ^,^ 75 ^,^ 76 The greatest risk is found in people reliant on insulin.^43^^,^ 71

*NCD-non specific*

Disasters can cause an exacerbation of NCDs or even death due to the limited access to treatment, care, medications and transport; lack of food and clean water; and increased exposure to extremes of cold or heat.^1^^,^ 76 ^,^ 77 ^,^ 78 ^,^ 79 ^,^ 80 ^,^ 81 ^,^ 82 ^,^ 83 Another risk factor is that people with NCDs are often evacuated without sufficient supplies of medication and pharmaceutical scripts/re-fills.^1^^,^ 74 ^,^ 84 ^,^ 85 ^,^ 86 It is common for evacuated people to no longer have access to the care they require.^78^ From a patient perspective, these factors often result in a perceived worsening of their condition, which can have negative impacts on their actual illness.^87^ For people with NCDs, a lack of treatment management and care for even a short period can result in severe exacerbations and preventable death.^1^^,^ 51 ^,^ 67 ^,^ 88 ^,^ 89

## Discussion

Cyclone, flood and cyclone related disasters interrupt treatment management and care for people with NCDs. This results in an increased risk of disease exacerbation or even death due to a range of factors, including damaged transport routes, unsafe water, reduced health services, loss of power and evacuations.^1^^,^ 9 ^,^ 42 ^,^ 46 ^,^ 47 ^,^ 49 ^,^ 50 ^,^ 52 ^,^ 59 ^,^ 62 ^,^ 64 ^,^ 65 ^,^ 67 ^,^ 69 ^,^ 70 ^,^ 76 ^,^ 77 ^,^ 78 ^,^ 80 ^,^ 81 ^,^ 82 ^,^ 83 ^,^ 86 A lack of appropriate care for even a short period of time puts the health and well-being of people with NCDs at risk.^90^^,^ 91 This is because people with NCDs are more vulnerable than others to the stresses and disruptions of a disaster.^81^

The impact of an interruption to treatment management and care varies according to the NCD. For people with cancer there is an increased risk of death, however, no evidence of a worsening in conditions.^45^^,^ 46 Cardiovascular incidents increase with exacerbations and complications such as higher blood pressure, heart attack and increased death rates.^46^^,^ 48 ^,^ 49 ^,^ 50 ^,^ 51 ^,^ 52 ^,^ 53 ^,^ 54 ^,^ 55 ^,^ 56 ^,^ 60 Chronic respiratory diseases are associated with acute exacerbation but no increase in death rates.^9^^,^ 52 ^,^ 59 ^,^ 66 People with diabetes experienced severe exacerbations, such as diabetic ketoacidosis, and an increased death rate with the risk greatest for insulin dependent diabetics.^1^^,^ 45 ^,^ 46 ^,^ 50 ^,^ 51 ^,^ 71 ^,^ 72 ^,^ 73

The reason for the variation in impact by NCD is due to the type of disruption to treatment management and care. For people with cancer, the increase in deaths can be associated with reduced access to transport, clinics, specialists, medications and hospitals.^42^^,^ 43 ^,^ 44 The situation for people with cardiovascular diseases is similar, however, delayed access to medical care and increased medication noncompliance are associated with exacerbations or even death.^49^^,^ 50 ^,^ 59 ^,^ 61 ^,^ 62 An exacerbation of a chronic respiratory disease is due to similar factors with a loss of power for equipment, such as nebulizers and oxygen, and mould growth additional issues.^52^^,^ 59 ^,^ 64 ^,^ 66 ^,^ 67 ^,^ 69 ^,^ 70 For people with diabetes an interruption to glucose monitoring, activity patterns and a loss of power for insulin storage increases the risk of exacerbation or even death.^43^^,^ 45 ^,^ 59 ^,^ 71 ^,^ 72 ^,^ 74 ^,^ 75 ^,^ 76

The long term health complications for people with NCDs requires further investigation. For example, uncontrolled diabetes for extended periods can result in heart disease (heart attacks and strokes), blindness, kidney failure and lower-extremity amputations.^92^^,^ 93 ^,^ 94 ^,^ 95 However, for diabetics and people with other NCDs there is limited data on the long term health complications associated with a disruption to treatment management and care due to a disaster. Understanding this, including the impact of reduced treatment efficacy, will provide a new paradigm for mitigating the impact of disasters on people with NCDs.

A challenge faced by this research and future studies is the range of descriptions for NCDs. NCDs are difficult to define because this group includes some diseases partly caused by infectious organisms (for example, cancers of the liver, stomach, and cervix) and usually excludes mental illnesses.^96^ When researching NCDs all variations (forms of the disease) should be considered, for example, chronic respiratory disease can include COPD and asthma. Of the NCDs subject to this research, cancer was the only condition where a single term can be used. It is recommended that future research focused on NCDs, as a collective, is guided by the major NCDs identified by WHO (cancer, cardiovascular, chronic respiratory and diabetes).

The review and subsequent analysis was limited to articles that predominately originated from high-income countries. Although this could be considered a limitation in the transferability of the findings, NCDs now disproportionately affect low and middle income countries.^97^^,^ 98 In African nations NCDs are rising rapidly and are projected to exceed communicable, maternal, perinatal, and nutritional diseases as the most common causes of death by 2030.^99^ However, caution should still be taken in applying the results to low and middle income countries as NCD priorities may change.

To mitigate the risk disasters pose to people with NCDs, a multi-sectoral approach is required. NCD treatment and care is reliant on more than just health services.^100^ For example, damage to transport routes prevents access to specialists, medications, nutritious food and health facilities and a loss of power is a threat for people reliant on electricity for insulin and oxygen.^42^^,^ 44 ^,^ 52 ^,^ 65 ^,^ 67 ^,^ 69 ^,^ 76 81 ^,^ 82 ^,^ 101 This interdependency highlights the need to mainstream health in disaster risk reduction activities at local, national and international levels.^101^

This research provides the platform required for expanding traditional disaster approaches by public health to incorporate NCDs. The reality of this need has been acknowledged in the Sendai Framework in the statement (30(k)) that chronic diseases (NCDs) need to be included in the design of disaster policies and plans.^3^ Specific measures may include cross-cutting disaster strategies such as multi-sectoral approaches to protect essential equipment/infrastructure, mapping health vulnerabilities within a community and stockpiling essential medicines.^102^^,^ 103 The aim is to ensure people with NCDs have access to life-saving services during and after disasters.^3^

To build on this research, the next step is to understand all the factors that influence both direct and indirect (preventable) morbidity and mortality related to NCDs during and after disasters. This includes quantifying acute complications, long-term complications and disease progression (including long term health complications and impacts of reduced treatment efficacy). Once achieved, disaster planners and public health professionals will be in a position to develop and implement effective and evidence-based mitigation strategies.

## Limitations

The research was influenced by the lead author’s studies and work in public health and disaster management in Australia at local, state and national levels and across the Asia-Pacific. To address this, an integrative methodology was selected to systematically identify and describe the literature. This included searching multiple databases and excluding grey and non-peer-reviewed literature. Papers were only selected if they were peer-reviewed and discussed cyclone, flood or storm related disasters and the impact on people with NCDs.

A limitation of this research is the narrow focus on the NCDs investigated: cancer; cardiovascular diseases; chronic respiratory diseases; and diabetes. This approach was selected to ensure consistency with the four major disease groupings for NCDs by the World Health Organization. However, other NCDs may be impacted more significantly by a disaster. For this reason caution should be taken in applying the results to other NCDs.

## Conclusion

Cyclone, flood and storm related disasters impact on treatment management and overall care for people with NCDs. This results in an increased risk of exacerbation of illness or even death. The interruption may be caused by a range of factors, such as damaged transport routes, reduced health services, loss of power and evacuations. The health impact varies according to the NCD. For people with chronic respiratory diseases, a disaster increases the risk of acute exacerbation. Meanwhile, for people with cancer, cardiovascular diseases and diabetes there is an increased risk of their illness exacerbating, which can result in death. To address this problem, there is a need to expand traditional disaster approaches by public health to incorporate NCDs. The reality of this need is further highlighted by the statements in the Sendai Framework. Specific measures may in include a multi-sectoral approach to ensure people with NCDs have access to life-saving services during and after disasters. To achieve this, the next step is to understand all the factors that influence both direct and indirect (preventable) morbidity and mortality related to NCDs during and after disasters. Once achieved, disaster planners and public health professionals will be in a position to develop and implement effective and evidence-based mitigation strategies.

## Human Participation Protection

Study protocol approval was not needed as there was no direct human participation in the study.

## Competing Interests

The authors have declared that no competing interests exist.

## Appendices

Appendix 1. PRISMA Checklist

Appendix 2. Individual Case Description
